# Paired-Associate and Feedback-Based Weather Prediction Tasks Support Multiple Category Learning Systems

**DOI:** 10.3389/fpsyg.2016.01017

**Published:** 2016-06-30

**Authors:** Kaiyun Li, Qiufang Fu, Xunwei Sun, Xiaoyan Zhou, Xiaolan Fu

**Affiliations:** ^1^State Key Laboratory of Brain and Cognitive Science, Institute of Psychology, Chinese Academy of SciencesBeijing, China; ^2^University of Chinese Academy of SciencesBeijing, China

**Keywords:** weather prediction task, feedback-based, paired-associate, non-declarative system, declarative system, task structure knowledge, self-insight knowledge

## Abstract

It remains unclear whether probabilistic category learning in the feedback-based weather prediction task (FB-WPT) can be mediated by a non-declarative or procedural learning system. To address this issue, we compared the effects of training time and verbal working memory, which influence the declarative learning system but not the non-declarative learning system, in the FB and paired-associate (PA) WPTs, as the PA task recruits a declarative learning system. The results of Experiment 1 showed that the optimal accuracy in the PA condition was significantly decreased when the training time was reduced from 7 to 3 s, but this did not occur in the FB condition, although shortened training time impaired the acquisition of explicit knowledge in both conditions. The results of Experiment 2 showed that the concurrent working memory task impaired the optimal accuracy and the acquisition of explicit knowledge in the PA condition but did not influence the optimal accuracy or the acquisition of self-insight knowledge in the FB condition. The apparent dissociation results between the FB and PA conditions suggested that a non-declarative or procedural learning system is involved in the FB-WPT and provided new evidence for the multiple-systems theory of human category learning.

## Introduction

It remains controversial whether probabilistic category learning can be mediated primarily by a non-declarative or procedural learning system (Poldrack et al., 2001; Newell et al., 2007; Poldrack and Foerde, 2008). To address this controversy, most studies have adopted a paradigm known as the weather prediction task (WPT), one of the most widely used paradigms in probabilistic category learning (e.g., Knowlton et al., 1994; Poldrack et al., 2001; Shohamy et al., 2004; Dickerson et al., 2011). As described in Knowlton et al. (1994), in the WPT, participants were given multidimensional stimuli (a set of four tarot cards as cues) and asked to classify those stimuli into one of two categories (rainy or sunny outcome); the cue-outcome associations are probabilistic, and participants can learn them through trial-by-trial feedback. This was termed the feedback-based WPT (FB-WPT). Some studies of clinical patients initially suggested that the FB-WPT recruited a non-declarative or procedural learning system (e.g., Knowlton et al., 1994; Poldrack et al., 2001). For example, it was found that people with amnesia exhibited normal learning performance on the FB-WPT but expressed poor explicit memory of this task, whereas people with Parkinson’s and Huntington’s disease showed impaired learning of this task but good explicit memory of the task features (Knowlton et al., 1996a,b). These findings provided evidence for the multiple category-learning systems assumption, which assumed that category learning is mediated by one declarative system and one non-declarative or procedural system (Zeithamova and Maddox, 2007). However, a growing body of evidence casts doubt on the recruitment of the non-declarative system in the FB-WPT (Lagnado et al., 2006; Newell et al., 2007; Price, 2009; Kemény and Lukács, 2013). For example, it has been demonstrated that participants reported accurate knowledge of both the task structure and their own judgment processes in the FB-WPT, indicating that the learning is mediated by one explicit declarative learning system (Lagnado et al., 2006).

An alternative method used to determine the recruitment of the non-declarative system in the FB-WPT is to compare this version with a paired-associate (PA) version, which is widely accepted to be dependent on the declarative learning system (Poldrack et al., 2001; Little and Lewandowsky, 2012). In the PA version, participants were presented simultaneously with the cue and the weather outcome, and were required to learn the associations in a PA manner. It should be noted that in both FB and PA versions, the associations between the cues and the outcomes are probabilistic, which was unknown to the participants (Poldrack et al., 2001). It was found that healthy participants in the FB version showed more activity in the caudate nucleus (part of the basal ganglia) and less activity in the medial temporal lobe (MTL), whereas participants in the PA version demonstrated the opposite pattern. Moreover, individuals with Parkinson’s disease and Huntington’s disease performed badly in the FB version due to basal ganglia dysfunction but did well in the PA version (Shohamy et al., 2004; Holl et al., 2012). These findings provided further evidence that learning probabilistic association in the FB-WPT employed the non-declarative learning system. However, these findings were not confirmed by behavioral analyses. Rather, it has been reported that there was no significant difference in the classification performance of healthy participants in the FB- and PA-WPT (Poldrack et al., 2001; Shohamy et al., 2004). Furthermore, Newell et al. (2007) demonstrated that participants displayed comparable and accurate insight into the tasks and their judgment processes in both the FB and PA versions of the WPT, indicating that learning in either version is mediated by one single explicit declarative learning system.

It has been found that the stimulus interval influences the acquisition of explicit knowledge in implicit sequence learning, such that the longer the learning time, the greater the tendency to acquire explicit sequence knowledge (Destrebecqz and Cleeremans, 2001, 2003; Cleeremans and Sarrazin, 2007; Fu et al., 2008). Previous empirical studies (e.g., Newell et al., 2007) using the FB-WPT extended the training time to 7 s for each trial, which may have enabled participants to consciously memorize certain classification rules (e.g., the comparable simple relationship between one card and the weather outcome) even in the FB version. Moreover, Knowlton et al. (1994) mentioned that while the FB-WPT is a somewhat non-motor implicit learning task that involves incremental learning over many trials, some declarative knowledge about the cue-outcome associations might develop after more extended training. If this is the case, shortening the training time might impair the acquisition of explicit knowledge in the FB-WPT, but it would have no effect on weather prediction performance. Conversely, as participants were asked to memorize the probabilistic association between the cue and the outcome as best as they could in the PA version, shortening the training time could impair both the categorization performance and the acquisition of explicit knowledge. To the best of our knowledge, no studies have directly tested the effects of training time in the FB and PA versions. Therefore, we manipulated the training time (7 or 3 s) in both FB and PA versions in Experiment 1 to investigate the role of training time in the different WPTs. We predicted that if this manipulation leads to apparent dissociations between the FB and PA versions, it will support the premise that the FB-WPT is mediated by a non-declarative or procedural learning system. However, the absence of dissociations may imply that there was no use of a non-declarative learning system in the FB version and the multiple category-learning systems must be reevaluated.

It has also been argued that the declarative system relies on verbal working memory while the non-declarative system does not (Filoteo et al., 2010; Lewandowsky et al., 2012). If learning in the FB and PA versions is mediated by different systems, the secondary verbal working memory task would have different effects in the two versions of the WPT. It has been found that the introduction of a numerical Stroop task in the FB version not only impaired learning but also reduced the number of participants relying on complex multi-cue strategies, thereby indicating that the FB version is mediated by the declarative system (Newell et al., 2007). Nonetheless, Miles and Minda (2011) noted that although the numerical Stroop task did have a verbal component (e.g., storing and rehearsing the information “big five, little seven”), it could be solved with a visual strategy; in which case, the impaired performance in the FB version could not support the purely declarative involvement in the FB task. Furthermore, it has been demonstrated that as a concurrent task, the Sternberg memory-scanning task impairs declarative category learning but not non-declarative category learning (Sternberg, 1966; Maddox et al., 2004; Zeithamova and Maddox, 2007). Thus, we added the Sternberg memory-scanning task in both versions in Experiment 2 to explore the role of verbal working memory in the FB- and PA-WPT when the training time for each trial was 7 s. We predicted that if the additional verbal working memory load selectively impairs learning in the PA version but not in the FB version, it would support the contention that the FB learning is mediated by a non-declarative system. However, if there are similar effects in the FB and PA versions, it would provide evidence for the single declarative learning system account.

## Experiment 1

### Materials and Methods

#### Participants

One hundred and four undergraduates were voluntarily recruited to participate in Experiment 1 and were randomly assigned to one of four groups. Each group was composed of 26 participants. None of the participants had previously participated in any probabilistic category-learning task. Experiments 1 and 2 were approved by the Institutional Review Board of the Institute of Psychology, Chinese Academy of Sciences. The data for six participants were excluded from the statistical analysis because the optimal accuracy was less than 50%. The data for 98 participants (44 female, *M* = 22.5, *SD* = 2.76) were included in the statistical analysis.

#### Apparatus and Stimuli

The stimuli were displayed on 17-inch cathode-ray tube (CRT) monitors with a refresh rate of 75 Hz and a screen resolution of 1024 × 768 pixels. The software package E-prime 2.0 was used for stimuli presentation and data collection.

In each version of the WPT, there were four cues (i.e., square, diamond, circle, and triangle tarot cards), and each cue was independently associated with each possible outcome (i.e., sunny or rainy) with a fixed probability (Knowlton et al., 1994). The square, diamond, circle and triangle cards predicted the outcome “sunny” with probabilities of 0.8, 0.6, 0.4, and 0.2, respectively, in the present study. In each trial, participants were presented with a particular combination of one, two or three of the four cards. There were 14 possible card combinations, as the patterns with or without all of the four cards were excluded. The card combinations were used to generate a series of 200 trials with different frequencies in which the two outcomes occurred equally often (see **Table 1**). The card or card combination appeared in a pseudo-random sequence with a limit that the same one did not appear twice in succession. In the training phase of the FB version, the line drawings of smiley and wrinkly cartoon faces were given as feedback indicating “correct” or “incorrect” after participants made their prediction. Meanwhile, the cumulative response accuracy was updated.

**Table 1:** Probabilistic structure of the weather prediction task.

Card patterns 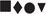	Probability		Frequency		Optimal response
	*P*(pattern)	*P* (sunny outcome)	Sunny	Total	
1 1 1 0	0.095	0.895	17	19	S
1 1 0 1	0.045	0.778	7	9	S
1 1 0 0	0.13	0.923	24	26	S
1 0 1 1	0.045	0.222	2	9	R
1 0 1 0	0.06	0.833	10	12	S
1 0 0 1	0.03	0.5	3	6	
1 0 0 0	0.095	0.895	17	19	S
0 1 1 1	0.095	0.105	2	19	R
0 1 1 0	0.03	0.5	3	6	
0 1 0 1	0.06	0.167	2	12	R
0 1 0 0	0.045	0.556	5	9	S
0 0 1 1	0.13	0.077	2	26	R
0 0 1 0	0.045	0.444	4	9	R
0 0 0 1	0.095	0.105	2	19	R

*Card patterns: 0 = card absent; 1 = card present. On any trial, one of the 14 possible combinations appeared with the probability P (pattern) indicated. Each combination of cards predicted sunny weather with a probability P (sunny outcome) and predicted rainy weather with a probability of 1 – *P* (rainy outcome). Optimal response: S, sunny weather; R, rainy weather.*

#### Design and Procedure

The experimental design consisted of a 2 (learning styles: FB vs. PA) ^∗^ 2(training time: 7 s vs. 3 s) between-subjects design. Each condition included a training phase, a testing phase, and an awareness testing phase.

##### The training phase

**FIGURE 1:**
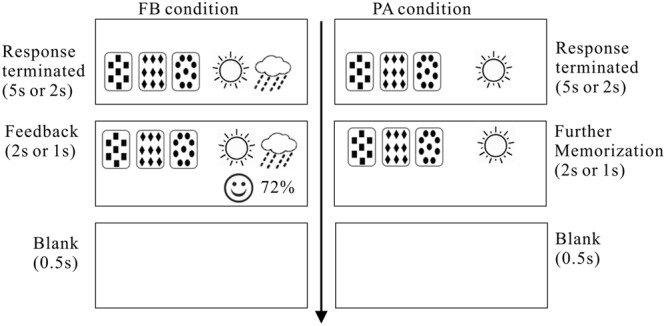
**Trial procedure of the training phase in Experiment 1.** FB refers to the feedback-based version, and PA refers to the paired-associate version.

In the FB condition, participants were instructed to forecast weather (sunny or rainy) based on the presented card or card combination. In each trial, one card or card combination was presented on the left screen and two outcomes were presented on the right screen (see **Figure 1**). The participants were asked to predict the weather by pressing the “F” or the “J” button within either a 5 s or a 2 s time period, depending on the condition. The positions of “sunny” or “rainy” were balanced between participants. If the participant did not respond within 3 or 1 s, depending on the condition, a prompt would be displayed that read, “Please answer as quickly as possible!” If there was still no answer within the next 2 or 1 s, the trial was terminated. If participants made the prediction in time, feedback was given at the bottom of the screen as a smiley face (correct) or wrinkly face (incorrect) and the cumulative response accuracy were updated. Participants were told that although they would guess the weather outcome randomly at the beginning, they would gradually improve their predictive performance through trial-by-trial feedback and eventually become good forecasters.

In the PA condition, a card or card combination and the corresponding weather outcome were presented simultaneously in each trial. Participants were given 5 or 2 s to memorize the association between the cue and the outcome. To be comparable with the FB condition, participants were asked to press the “F” button or the “J” button within 5 or 2 s while keeping in mind the association between the cue and the outcome. If the participant did not press either the “F” or “J” button within 3 or 1 s, a prompt of “Please press as quickly as possible” would be displayed. After each response, an additional 2 or 1 s time period was given for participants to further memorize the cue and the outcome.

Participants were not told about the exact cue value and probabilistic relationship in either the FB or the PA version. Each condition consisted of 200 trials in the training phase.

##### The testing phase

After the training phase, participants had a minimum of 60 s to rest before being asked to complete the testing phase. In the testing phase, participants were required to predict the weather based on what they learned in the training phase. In each trial of both the FB and PA conditions, a card or card combination was presented and participants were asked to predict weather by pressing either the “F” or the “J” button within 5 or 2 s, which is consistent with the conditions in the training phase. The response buttons were balanced between participants. After the prediction, no feedback was given, and there was no additional memorization time. Each condition also consisted of 200 trials in the testing phase.

##### The awareness testing phase

After the testing phase, we adopted the measures used by Lagnado et al. (2006) and Wilkinson et al. (2008) to assess the conscious status of the two types of knowledge. In the probability test, which measured the conscious status of task-structure knowledge, participants were asked to rate the probability of sunny versus rainy weather for each of the four cards. Participants indicated their responses on a continuous sliding scale from 0 to 100, where 0 = definitely rainy, 50 = equally likely to be rainy or sunny, and 100 = definitely sunny. In the importance test, which measured the conscious status of self-insight knowledge, participants were asked to indicate the importance of the square/circle/diamond/triangle card in their decision of the outcome during the testing phase. Responses were indicated on a continuous sliding scale from 0 to 100, where 0 = not important at all, 50 = moderately important, and 100 = very important.

### Results

#### The Optimal Accuracy

**FIGURE 2:**
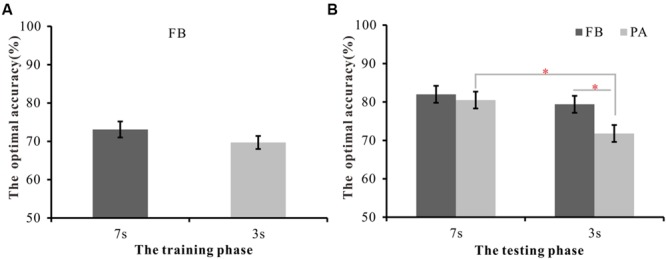
**The mean optimal accuracy of the training and testing phases for each condition in Experiment 1. (A)** The optimal accuracy of the training phase for 7 and 3 s FB-WPT conditions. **(B)** The optimal accuracy of the testing phase under 7 and 3 s FB-WPT as well as those for PA-WPT conditions. FB refers to the feedback-based version, and PA refers to the paired-associate version. Error bars represent 2 SEM. ^∗^*p* < 0.05, ^∗∗^*p* < 0.01, ^∗∗∗^*p* < 0.001.

**Figure 2** depicts the mean optimal accuracy on the FB-WPT in the training phase and the mean optimal accuracy on the FB- and PA-WPT in the testing phase. The optimal accuracy in the training and testing phases were analyzed as in Gluck et al. (2002). To compare the optimal accuracy for different training times in the training phase of the FB version, an independent *t*-test was used. That analysis revealed no significant difference between the 7 s (*M* = 73.1%) and 3 s training time conditions (*M* = 69.7%) in the FB task [*t*(47) = 1.25, *p* = 0.22].

To explore the effect of training time in the FB- and PA-WPTs, a 2 (learning style: FB vs. PA) ^∗^ 2 (training time: 7 s vs. 3 s) between-subjects ANOVA on the optimal accuracy in the testing phase was used. It revealed a significant learning style effect [*F*(1,94) = 4.19, *p* < 0.05, $\eta_{p}^{2}$ = 0.043], with better performance in the FB version (80.7%) than that in the PA version (76.1%). A significant training time effect was also observed [*F*(1,94) = 6.47, *p* < 0.05, $\eta_{p}^{2}$ = 0.064], with higher optimal accuracy corresponding to longer training trials (81.2%) rather than to shorter training trials (75.6%). The interaction did not reach significance [*F*(1,94) = 1.68, *p* = 0.17]. Nonetheless, the planned contrasts revealed that there was no significant difference in the optimal accuracy between the 7 s (81.9%) and 3 s (79.4%) trials in the FB version [*t*(47) = 0.91, *p* = 0.34], but the optimal accuracy in the 7 s trials (80.5%) was significantly higher than that in the 3 s trials (72.1%) in the PA version [*t*(47) = 5.66, *p* < 0.05, *d* = 0.68]. Furthermore, no significant difference between FB and PA conditions was found when the training time was 7 s [*t*(47) = 0.29, *p* = 0.59], whereas performance on the FB version was significantly higher than performance on the PA version when the training time was 3 s [*t*(47) = 4.83, *p* < 0.05, *d* = 0.60]. These results suggest that while shorter training time can significantly decrease the PA learning performance, it does not decrease FB learning performance.

#### Awareness Knowledge

##### Task knowledge

**FIGURE 3:**
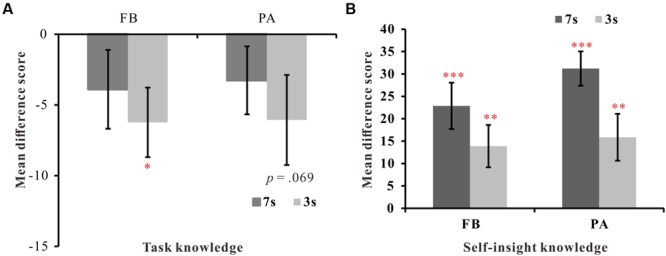
**Mean difference scores of probability ratings or importance ratings for each condition in Experiment 1. (A)** Mean difference scores between probability ratings and the actual probabilities for each condition in Experiment 1. **(B)** Mean difference scores between importance ratings for strong and weak cards for each condition in Experiment 1. FB refers to the feedback-based version, and PA refers to the paired-associate version. Error bars represent 2 SEM. ^∗^*p* < 0.05, ^∗∗^*p* < 0.01, ^∗∗∗^*p* < 0.001.

To estimate how well the participants’ ratings matched the actual card probabilities, we first calculated the difference score for each participant by subtracting the actual card probability (i.e., 0.2, 0.4, 0.6, and 0.8) from the ratings for each card. We then calculated the mean difference scores of the four cards and compared the mean difference scores with zero using a one-way simple *t*-test. If there was no significant difference between the mean score and zero, it indicated that participants were extremely accurate in their probability judgments. Conversely, a significant positive or negative difference between the mean score and zero was indicative of an overestimation or underestimation of the actual probabilities. This method has been used by Wilkinson et al. (2008). **Figure 3A** presents the mean difference score for each condition. When training time was 7 s, the participants’ mean difference scores on the two learning versions were not significantly different from zero [*t*(23)_FB_ = -1.39, *p* = 0.17, *t*(24)_PA_ = -1.32, *p* = 0.20], thus indicating that all participants were accurate in their probability judgments. However, when the training time was shortened to 3 s, participants in the FB condition significantly underestimated the actual probabilities of the cues [*t*(24) = -2.59, *p* < 0.05, *dz* = 1.04], whereas participants in the PA condition also underestimated the actual probabilities of the cues [*t*(23) = -1.90, *p* = 0.069, *dz* = 0.78] to some extent. Moreover, a 2 (learning style: FB vs. PA) ^∗^ 2 (training time: 7 s vs. 3 s) between-subjects ANOVA revealed no significant main effects or interaction effect. These results suggest that a shorter training time may have impaired the acquisition of explicit task structure knowledge, especially in the FB condition.

##### Self-insight

To explore whether participants could discriminate strongly predictive cards (square and triangle card) from weakly predictive cards (diamond and circle card); we first combined the ratings for the two strongly predictive cards into one group (the strong group) and the ratings for the two weakly predictive cards into another group (the weak group). We then calculated the mean difference scores between the strong and weak group for each participant and compared the mean difference score with zero using a one-way simple *t*-test. No significant difference or a significant negative difference between the mean score and zero indicated the participants’ poor abilities to differentiate between the strong and weak cards, whereas a significant positive difference score was indicative of good self-insight knowledge. **Figure 3B** presents the mean difference scores between strong and weak cards in each condition. The one-way *t*-test revealed that all participants in the four conditions differentiated strong from weak cards very well [*t*(23)_FB-7s_ = 4.41, *p* < 0.001, *dz* = 1.82; *t*(24)_PA-7s_ = 6.60, *p* < 0.001, *dz* = 2.63; *t*(24)_FB-3s_ = 3.62, *p* < 0.01, *dz* = 1.51; and *t*(23)_PA-3s_ = 3.02, *p* < 0.01, *dz* = 1.24]. Furthermore, a 2 (learning style: FB vs. PA) ^∗^ 2 (training time: 7 s vs. 3 s) ANOVA performed on the mean difference scores revealed only a significant training time effect [*F*(1,94) = 6.54, *p* < 0.05, $\eta_{p}^{2}$ = 0.065], thus indicating that participants were better able to differentiate the strong and the weak cards when the training time was 7 s (27.05) compared to when the training time was 3 s (14.87). No other significant effects or interactions were found.

### Discussion

The results of Experiment 1 indicated that participants’ optimal accuracy in the PA condition was significantly decreased when the training time was reduced from 7 to 3 s, when they had inaccurate knowledge of the task structure and there was a decline in their ability to distinguish between strong and weak cards. However, although shortening the training time led to inaccurate task structure knowledge of participants in the FB condition, this lack of training time did not affect participants’ optimal accuracy with respect to the FB-WPT, neither in the training phase nor in the testing phase. Thus, these results suggest that participants could learn how to predict without awareness in the FB version. The apparent discrepancies between the PA and FB versions were inconsistent with the assertion of Newell et al. (2007), who suggested that there was only one single explicit or declarative learning system. Rather, the results were consistent with the assumption of multiple learning system theory.

## Experiment 2

### Materials and Methods

#### Participants

Fifty-two college students participated in Experiment 2. None of the participants had previously participated in a probabilistic category-learning task. The data of seven participants were excluded from further analysis because the concurrent task performance was less than 90%. The data of 45 participants (25 female, *M* = 22.1, *SD* = 2.02) were therefore included in the statistical analysis.

#### Materials

The stimuli in the FB and PA versions were identical to those used in Experiment 1. The digits of the Sternberg memory-scanning task included ten numbers that appeared in 44-point black font.

#### Design and Procedure

As in Experiment 1, each condition included a training phase, a testing phase, and an awareness-testing phase. During the training phase, the procedure for the WPT was embedded in the Sternberg memory-scanning task and was identical to that used in the FB and PA versions with 7 s training times in Experiment 1. In each trial, four digits were first displayed in the center of the screen for 500 ms, and participants were asked to remember the four digits. The four digits were randomly selected from 0 to 9 without replacement. Then, participants were asked to finish the WPT task depending on the instructions of the FB and PA version. Finally, a single digit was presented on the screen along with the question, “Was this digit originally shown?” In half of the cases, the digit was one of the four initial digits, whereas in the other half of the cases, it was not. Participants responded by pushing the “F” key or the “J” key on the keyboard, which indicated a “yes” or “no” response, respectively. The positions for “yes” and “no” were counterbalanced between participants. To encourage participants to do as well as possible on the concurrent task, they would receive a warning, “Please try your best to remember the initial four digits,” when they responded incorrectly twice during the digit task. Each condition consisted of 200 trials in the training phase. The testing phase and the awareness-testing phase were identical to the 7 s conditions in Experiment 1.

### Results

#### Concurrent Task Performance

There were no significant differences regarding the mean accuracy of the concurrent task between the FB and PA conditions [*t*(43) = -0.005, *p* = 0.99]. Moreover, there was no significant difference in the response times between the two conditions [*t*(43) = -0.40, *p* = 0.69]. This result indicates that if there was a different effect of the concurrent task in the two conditions, it could not be attributed to the effect of the concurrent task itself.

#### The Optimal Accuracy

**FIGURE 4:**
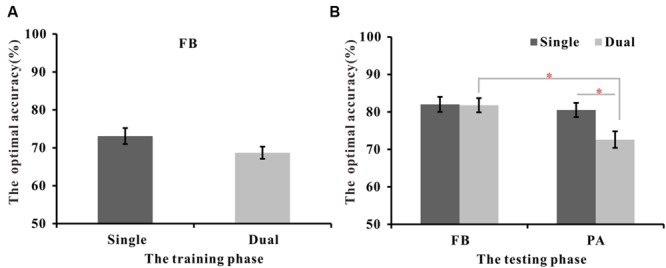
**The mean optimal accuracy of the training and testing phases for the dual and single conditions. (A)** The optimal accuracy of the training phase under single and dual FB-WPT conditions. **(B)** The optimal accuracy of the testing phase for single and dual FB-WPT as well as that for PA-WPT conditions. FB refers to the feedback-based version, and PA refers to the paired-associate version. Error bars represent 2 SEM. ^∗^*p* < 0.05, ^∗∗^*p* < 0.01, ^∗∗∗^*p* < 0.001.

With the exception of a concurrent task, the WPT in Experiment 2 was identical to the FB and PA versions with 7 s training times used in Experiment 1; thus, we called the WPT task in Experiment 2 the dual-FB or dual-PA task and the corresponding task in Experiment 1 the single-FB or single-PA task. **Figure 4** shows the optimal accuracy in the training phase and the testing phase for the dual- and single-task conditions. An independent-samples *t*-test on the optimal accuracy in the training phase in the FB version revealed that there was no significant difference between the single condition (*M* = 73.1%) and the dual condition [*M* = 68.7%; *t*(47) = 1.68, *p* = 0.10].

The optimal accuracy during the testing phase in the dual-FB condition (*M* = 81.8%) was significantly higher than those in the dual-PA condition [*M* = 72.5%; *t*(43) = 3.03, *p* < 0.01, *d* = 0.91]. To further examine the effect of the concurrent verbal task on the FB and PA versions, a 2 (learning style: FB vs. PA) ^∗^ 2 (task type: single vs. dual) ANOVA was used. It revealed a significant learning style effect [*F*(1,90) = 7.11, *p* < 0.01, $\eta_{p}^{2}$ = 0.073], a significant task type effect [*F*(1,90) = 4.08, *p* < 0.05, $\eta_{p}^{2}$ = 0.043], and a marginally significant interaction effect [*F*(1,90) = 6.94, *p* = 0.057, $\eta_{p}^{2}$ = 0.04]. The planned contrast revealed that there was no significant difference in the optimal accuracy in the single- and dual-FB conditions [*t*(47) = 0.07, *p* = 0.94], but the performance in the dual-PA condition was significantly decreased compared to the single-PA condition [*t*(43) = 2.58, *p* < 0.05, *d* = 0.77]. That is, the PA learning, but not the FB learning, was impaired by the additional secondary load.

#### Awareness Knowledge

##### Task knowledge

**FIGURE 5:**
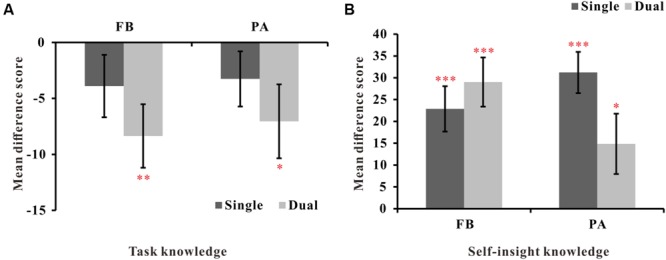
**Mean difference scores of probability ratings or importance ratings for dual and single conditions. (A)** Mean difference scores between probability ratings and the actual probabilities for dual and single conditions. **(B)** Mean difference scores between importance ratings for strong and weak cards for dual and single conditions. FB refers to the feedback-based version, and PA refers to the paired-associate version. Error bars represent 2 SEM. ^∗^*p* < 0.05, ^∗∗^*p* < 0.01, ^∗∗∗^*p* < 0.001.

To estimate how well the participants’ ratings matched the actual card probabilities in the dual conditions, we calculated the mean difference scores as in Experiment 1. **Figure 5A** presents the mean difference scores of the FB and PA versions in single and dual conditions. One-way simple *t*-tests revealed that participants in the dual FB and PA conditions significantly underrated the actual predicative probabilities [*t*(24)_FB_ = -2.94, *p* < 0.01, *dz* = 1.18, *t*(19)_PA_ = -2.13, *p* < 0.05, *dz* = 0.96, respectively], thus indicating that the participants did not acquire accurate explicit knowledge about the predictive probability of the cards. Further, a 2 (learning style: FB vs. PA) ^∗^ 2 (task type: single vs. dual) between-subjects ANOVA revealed no significant differences in the card rating scores across the training time condition or between single and dual learning groups.

##### Self-insight

To explore whether participants could discriminate between strongly predictive cards and weakly predictive cards, we calculated the mean difference scores between strongly predictive cards and weakly predictive cards. **Figure 5B** presents the mean difference scores in the dual and single conditions. One-way simple *t*-tests revealed that participants could significantly differentiate between strong and weak cards in the dual-FB [*t*(24) = 5.17, *p* < 0.001, *dz* = 2.14] and dual-PA conditions [*t*(19) = 2.15, *p* < 0.05, *dz* = 0.96]. Furthermore, a 2 (learning style: FB vs. PA) ^∗^ 2 (task type: single vs. dual) ANOVA revealed a significant interaction effect [*F*(1,90) = 4.07, *p* < 0.05, $\eta_{p}^{2}$ = 0.04]. A simple effect analysis showed that there were no significant differences in the mean difference scores between the single- and dual-FB versions (*p* = 0.43), whereas the mean difference score of the dual-PA was significantly decreased compared to the single-PA version (*p* < 0.05). No main effects of learning style and task type were found (*F*s < 1). These results suggest that the concurrent task impaired the acquisition of explicit knowledge about self-insight in the PA version but not in the FB version.

### Discussion

The results in Experiment 2 revealed that compared with the single PA condition in Experiment 1, the four-digit memory scanning task diminished the optimal accuracy in the dual-PA condition and impaired the participants’ knowledge acquisition about the task structure and about self-insight. However, compared with the single FB condition in Experiment 1, the concurrent task did not impair the optimal accuracy and self-insight in the dual-FB condition. The different effects of the concurrent task in the PA and FB versions indicate that different cognitive learning systems are involved in the PA and FB versions of the WPT.

## General Discussion

Although the FB and PA versions of the WPT share the common goal of learning the value of probabilistic cues (Dickerson et al., 2011), the PA-WPT is widely accepted to recruit the declarative learning system, whereas whether the FB-WPT employs a non-declarative or procedural learning system remains controversial. Thus, to investigate whether probabilistic category learning can be dependent on a non-declarative or procedural learning system, we compared the effects of training time and verbal working memory that influence the verbal learning system but not the non-verbal learning system in the FB-WPT and the PA-WPT. Our results revealed that the manipulation of training time and verbal working memory influenced optimal accuracy in the PA version but not in the FB version. The apparent dissociations of optimal responses between the two versions suggested that the FB-WPT is mediated by a non-declarative or procedural learning system.

Specifically, in Experiment 1, we found that when the training time was 7 s, there were no significant differences between the FB and PA versions in either the optimal accuracy or the awareness test performance. These results were consistent with the findings of Newell et al. (2007), which supported the single explicit learning system account. However, when the training time was reduced to 3 s, the optimal accuracy was higher in the FB version than in the PA version, as the performance was not reduced in the FB version but was significantly impaired in the PA version. Moreover, although shortening the training time decreased the expression of explicit knowledge in both conditions, only participants in the FB condition expressed significantly implicit knowledge about the task structure. These findings were principally consistent with the findings of Poldrack et al. (2001) and Shohamy et al. (2004), which support the multiple learning system account. Thus, as shortening the training time led to the apparent dissociations between the FB and PA versions, our results suggest that the FB-WPT can be mediated by a non-declarative or implicit learning system.

Interestingly, though Knowlton et al. (1994) once mentioned that extended training in FB-WPT might lead to the development of declarative knowledge about the cue-outcome associations, the FB version is typically used as an implicit non-declarative task. Coincidently, Destrebecqz and Cleeremans (2001, 2003) further assumed that the development of explicit knowledge takes time, and a number of studies have shown that time access determines whether the memory traces are available for verbal reporting and cognitive control (Ashby et al., 2002; Edmunds et al., 2015). To the best of our knowledge, our study is the first to demonstrate that limiting the training time impairs the acquisition of explicit knowledge but not the expression of implicit knowledge in the FB-WPT.

In Experiment 2, we found that the optimal accuracy was consistently significantly higher in the dual-FB condition than it was in the dual-PA condition because the concurrent four-digit memory-scanning task diminished performance in the PA condition but not in the FB condition. Interestingly, the concurrent task reduced the acquisition of explicit knowledge in both conditions, especially in the FB condition. Consistently, Foerde et al. (2006, 2007) found that a concurrent tone-counting task did not reduce performance in the FB task but did reduce the amount of declarative learning about the task, especially in the FB condition. As the declarative system relies on verbal working memory whereas the non-declarative system does not, the different roles of the concurrent working memory task in the FB and PA versions indicate that learning in the FB and PA versions of the WPT depends on qualitatively distinct cognitive learning systems.

It should be noted that our results of the dual-FB version are inconsistent with the findings of Newell et al. (2007), who found that the addition of a concurrent numerical Stroop task reduced the learning performance and the number of participants relying on complex multi-cue strategies. The reason for this discrepancy might be that the numerical Stroop task used in Newell et al. (2007) impaired not only declarative learning but also non-declarative learning because people can solve the numerical Stroop task with a visual strategy, whereas the Sternberg memory-scanning task impairs only declarative category learning but not non-declarative category learning. Further research must directly compare the roles of the two types of dual tasks in the FB-WPT and PA-WPT.

Previous studies have demonstrated that there is dissociation between performance on the FB-WPT and self-reported knowledge about the task (e.g., Gluck et al., 2002; Evans et al., 2003). This dissociation has been viewed as evidence of the multiple learning system account. However, when two more sensitive measures of explicit knowledge are adopted, i.e., the probability test and the importance-rating test, no such dissociation is found for the healthy participants in the FB-WPT (e.g., Lagnado et al., 2006; Newell et al., 2007). Nonetheless, studies of clinical patients and the neural imaging studies of healthy participants have revealed that FB learning is mediated by basal ganglia, including the caudate nucleus and the striatum, which contribute to non-declarative memory (Packard and Knowlton, 2002; Shohamy et al., 2004). Therefore, our study extends these findings and further shows that healthy participants can acquire implicit knowledge in the FB-WPT when the training time is shortened or a concurrent verbal working task is added, thus providing new evidence for the multiple learning system account.

Furthermore, the neural underpinnings of FB learning may elucidate the robust performance on the FB-WPT compared with that on the PA-WPT. It has been found that neural circuitry implicated in the incremental learning of the FB-WPT is also involved in reward prediction and that a reward-mediated feedback signal within the basal ganglia is provided by the release of dopamine from the substantia nigra (Shohamy et al., 2008). Specifically, dopamine is released into the tail of the caudate from the substantia nigra shortly after the observer receives an unexpected reward; the presence of this dopamine is widely thought to strengthen recently active synapses (Wickens, 1990; Ashby et al., 2002). Thus, it is possible that once the feedback is given in the FB learning, the association between the cue and the outcome is strengthened regardless of the training time or verbal working memory load. However, this proposition is tentative and requires further investigation.

As the training time and the verbal working memory load play different roles in the FB and PA versions, the results of the present study suggest that a non-declarative learning system is used in learning the probabilistic associations between cues and weather outcomes in the FB-WPT. These results lend further support to the hypothesis that human probabilistic category learning is mediated by multiple systems.

## Author Contributions

KL and QF designed the experiment and wrote the manuscript, KL and XS performed the experiment and analyzed the collecting data, XS, XZ, and XF revised the manuscript.

## Conflict of Interest Statement

The authors declare that the research was conducted in the absence of any commercial or financial relationships that could be construed as a potential conflict of interest.
